# Phosphoregulation of Kinesins Involved in Long-Range Intracellular Transport

**DOI:** 10.3389/fcell.2022.873164

**Published:** 2022-06-03

**Authors:** Diksha Kumari, Krishanu Ray

**Affiliations:** Department of Biological Sciences, Tata Institute of Fundamental Research, Mumbai, India

**Keywords:** Kinesin, kinase, phosphorylation, axonal transport, vesicle traffic, regulation

## Abstract

Kinesins, the microtubule-dependent mechanochemical enzymes, power a variety of intracellular movements. Regulation of Kinesin activity and Kinesin-Cargo interactions determine the direction, timing and flux of various intracellular transports. This review examines how phosphorylation of Kinesin subunits and adaptors influence the traffic driven by Kinesin-1, -2, and -3 family motors. Each family of Kinesins are phosphorylated by a partially overlapping set of serine/threonine kinases, and each event produces a unique outcome. For example, phosphorylation of the motor domain inhibits motility, and that of the stalk and tail domains induces cargo loading and unloading effects according to the residue and context. Also, the association of accessory subunits with cargo and adaptor proteins with the motor, respectively, is disrupted by phosphorylation. In some instances, phosphorylation by the same kinase on different Kinesins elicited opposite outcomes. We discuss how this diverse range of effects could manage the logistics of Kinesin-dependent, long-range intracellular transport.

## Introduction

Kinesins represent a superfamily of mechanochemical enzymes that bind to microtubules, hydrolyze ATP and either transport cargoes along the microtubule tracks or participate in controlling microtubule dynamics inside a cell (Vale and Milligan, 2000; Hirokawa and Noda, 2008). All Kinesins have three consensus structure-function domains engaged in motor activity (head domain), dimerization (stalk domain), and cargo binding (tail domain). Motors of the Kinesin-1, -2 and -3 families are primarily involved in long-range intracellular transport towards the cell periphery and synapse (Box 1) (Lawrence et al., 2004; Hirokawa and Noda, 2008). They contribute to one of the fundamental aspects of cellular functions–the logistics of moving molecular complexes and organelles from one point to another inside a cell (Ray, 2006). Kinesins have two primary biochemical functions—1) the microtubule-dependent ATPase activity that generates the mechanical force along the microtubule (Vale and Milligan, 2000; Qin et al., 2020), and 2) reversible interaction with soluble and vesicle-associated/transmembrane proteins, and membrane lipids (Hirokawa and Noda, 2008). Synchrony between these two actions leads to an effective movement of proteins, vesicles and organelles inside a cell (Akhmanova and Hammer, 2010).

Kinesin motor activity, cargo-binding and the flux of cargo transport driven by the motor are tuned according to the cargo availability and cellular environments. Motor activation and spatiotemporally modified cargo affinity are the two aspects of this regulation. An intramolecular interaction strategy, known as autoinhibition, blocks the ATPase activity when the motor is not associated with a cargo (Box 1). For some Kinesins in the auto-inhibited state, a part of the “tail” domain binds to the “motor” domain, preventing the ATP-ADP exchange (Hackney and Stock, 2000). A similar inhibition occurs through a motor-stalk interaction in the case of a Kinesin-3 family motor (Siddiqui and Straube, 2017). In both cases, cargo binding releases the inhibition. In addition, the start and stop signals, the direction and the speed of cargo traffic driven by motor proteins are actively controlled at multiple levels (Koppers and Farías, 2021; Guedes-Dias and Holzbaur, 2019). Amongst several other factors, phosphorylation of kinesins and their adaptors plays a crucial role in this process.

Phosphorylation-dephosphorylation cycles brought upon by kinases and phosphatases, respectively, downstream of signaling receptors are the most widely utilized mechanisms of global systems management inside a cell. The process helps coordinate a vast array of chemical reactions in functionally correlated subcellular compartments and conveys the effects of external stimuli to internal targets and *vice versa*. The information is relayed by cytoskeleton—associated motor proteins, which move the signaling complexes from their sources to the destinations inside a cell (Liang and Yang, 2019; (Bhabha et al., 2016; Akhmanova and Hammer, 2010). Several studies suggested that the kinases and phosphatases engaged in the signal transduction also act on molecular motors regulating the logistics of this information flow.

This review discusses the effects of site-specific phosphorylation on kinesin motility and intracellular transport. We describe the effects of phosphorylation on motor function and cargo binding and how phosphorylation orchestrates the logistics of kinesin-driven traffic inside a cell. A large body of literature, as summarized below, suggests that a select group of kinases (Box 3) also controls the logistics of relatively long-range transport carried by Kinesins 1–3. The phosphorylation sites, located in the motor domain, stalk-tail domains, or non-motor accessory subunits (Table 1), are unique to each Kinesin family, and some of them are only partially conserved. The results indicate that each phosphorylation imparts a unique effect. Motor activation and modification of various cargo-motor interactions are the most widely reported effects of kinesin phosphorylation. Besides, phosphorylation of an extensive repertoire of kinesin adaptors also regulates cargo attachment (Table 2). In many cases, the cargo association *de facto* activates the motor function, indicating the dual impact of this action. Therefore, the data is discussed according to the motor type and adaptors.

**TABLE 1:** List of kinases that phosphorylate kinesins.

Kinesin	Subunit	Kinase	Residue	Effect	Reference
Kinesin-1	DmKHC	GSK3β	S314	Attenuates motor activity; reduces run length and velocity	Banerjee et al. (2021)
	HsKHC	JNK	S175	Stabilizes the folded conformation of kinesin	Padzik et al. (2016)
	HsKHC, HsKLC	PKA	unknown	Releases kinesin from the synaptic vesicles	DeBerg et al. (2013) Sato-Yoshitake et al. (1992)
	HsKHC, HsKLC	PKC	unknown	Increases the ATPase activity Increases the ATPase activity	Matthies et al. (1993)
	HsKLC	GSK3β	S615	Releases membrane-bound organelles at growing neurite tips	Morfini et al. (2002)
	HsKLC1	ERK	S460	Weakens kinesin-1 interaction with Calsyntenin-1	Vagnoni et al. (2011)
	HsKLC1	PKC?	T466	Inhibits KLC1-JIP1 interaction	Chiba et al. (2017)
	MmKLC2	unknown	S575	Promotes 14-3-3 η binding	Ichimura et al. (2002)
	MmKLC2	AMPK	S539 and S575	Regulates the interaction of Kinesin-1 with regulatory subunit p85 of PI3K	Amato et al. (2011)
	CeKLC	CamKII	S240, S276	Promotes GLR-1 transport	Hoerndli et al. (2015)
Kinesin-2	MmKIF3A	CaMKII	S689	Recruitment of N-cadherin to KIF3A	Ichinose et al. (2015)
	MmKIF3A	CILK/ICK	T674	Ciliary length regulation by regulating the handover between anterograde and retrograde IFT machinery at ciliary tip	Chaya et al. (2014)
	CrFla8 (KIF3B)	CaMKII	S663	Releases IFT-B particles at the tip of regenerating flagella	Liang et al. (2014)
	MmKIF17	CaMKII	S1029	Dissociation of KIF17 from its adaptor Mint1/Lin10	Guillaud et al. (2008), Yin et al. (2012)
				Enhances KIF17 localization in photoreceptor outer segment	
	HsKAP3	SFK	unknown	Weakens the affinity with SmgGDS	Shimizu et al. (1996)
	MmKAP3A	MARK/Par1b	S60	Decreases the association between KAP3 and TRIM46	Ichinose et al. (2019)
Kinesin-3	KIF1c	CK2	S1092	Binding with 14-3-3 proteins	Dorner et al. (1999)
	MmKIF1A	CaMKII	S1665	Increases association of KIF1a with DCV	Hummel and Hoogenraad. (2021)
	KIF1A	GSK3β	S402	unknown	Gan et al. (2020)
	KIF13B (rat)	CDK5	T506	TRPV receptor binding	Lu and Prehoda. (2013)
	HsKIF13B	MARK/Par1b	S1381, S1410	promote 14-3-3 *ß* binding and intramolecular interactions in KIF13B inhibiting its interaction with microtubules	Yoshimura et al. (2005)
	DmKHC73	MARK/Par1b	S1374	binds to 14-3-3 ζ and helps in mitotic spindle organization	Lu and Prehoda. (2013)

**TABLE 2:** List of phosphorylation targets of kinesin adaptors.

Adaptor	Kinase	Target site	Effect	References
JIP1 (Mouse)	JNK	S421	Increases its binding to KHC	Fu and Holzbaur. (2013)
Aplip1/JIP1 (*Drosophila*)	Wnd/MAPKKK, Hep/MAPKK	unknown	Inhibits Aplip1/JIP-KLC binding	Horiuchi et al. (2007)
Fez1 (Human)	MARK2	S58	Promotes FEZ1-mediated transport; prevents aggregation of FEZ1 vesicles	Butkevich et al. (2016)
Fez1 (*Drosophila*)	Unc51/Atg1	S143	Increases its binding to Synaptotagmin-1	Toda et al. (2008)
Alcn-α/Clstn-1	CK1/CK2	Multiple serine residues	Enhance its interaction with KLC	Sobu et al. (2017)
CRMP2	GSK3β	T514	Reduces its binding to Tubulin	Yoshimura et al. (2005)

## Effects of Kinesin-1 Phosphorylation on Motor Function

One of the earliest *in vitro* studies suggested that bovine Kinesin-1 could be phosphorylated by cyclic-Adenosine Monophosphate (cAMP)-dependent protein kinase (cAMP-PK/PKA), protein kinase C (PKC) and pp60c-src (Matthies et al., 1993). Amongst these, PKA phosphorylated at multiple sites of KLCs, whereas the PKC phosphorylated both KHC and KLCs. The PKA-dependent phosphorylation enhanced the microtubule-stimulated ATPase activity, i.e., the motor function, of purified Kinesin-1 (Matthies et al., 1993). Also, KLC phosphorylation by a 100 kDa kinase copurified with Kinesin-1 holoenzyme increased the microtubule-dependent ATPase activity and microtubule gliding by the motor *in vitro* (McIlvain et al., 1994; Lindesmith et al., 1997). Although the target sites of these kinases on Kinesin-1 were not identified, these early studies suggested that phosphorylation could control kinesin-dependent intracellular transport.

Subsequently, two independent studies further indicated that phosphorylation of the KHC motor domain would disrupt motor activity (Table 1), and the incidents were linked to specific neurodegenerative disorders. For instance, increased activity of the poly-Q expanded Androgen Receptor (AR), which causes Spinal and Bulbar Muscular atrophy (SBMA), arrested Kinesin-1 motility and fast axonal transport by phosphorylating KHC through downstream activation of cJun N-terminal Kinase (JNK) (Morfini et al., 2006). Similarly, JNK3 activation due to the expression of pathogenic huntingtin protein phosphorylates a conserved serine residue (S175) of mouse Kif5A (Morfini et al., 2009a; Morfini et al., 2009b). Phosphorylation of an equivalent serine residue of Kif5B attenuated the load-bearing capacity of the motor, biasing a minus-end directed cargo transport *in vitro* and stabilized the autoinhibited conformation (DeBerg et al., 2013). A separate study further suggested that although the JNK phosphorylation of an equivalent S176 residue of mouse Kif5C could enhance the ATPase rate, it significantly reduced the microtubule affinity *in vitro* and stalled the movement of kinesin-associated vesicles in the axon (Padzik et al., 2016). Altogether, the data suggest that S175/176 phosphorylation stabilizes an autoinhibited conformation of Kinesin-1 and attenuates the load-bearing capacity of the motor in the absence of cargo *in vitro* and enhances the minus-end-directed vesicle movement *in vivo*. The S175/176 residue is located in the loop8-β5 region of the kinesin motor domain involved in microtubule binding (Woehlke et al., 1997). The structure of this region displays considerable variation amongst different types of kinesins (Scarabelli and Grant, 2013). This insight led us to conjecture that increasing the negative charge in this loop could alter the microtubule affinity.

A recent study further suggested that Glycogen Synthase Kinase 3β (GKS3β) could phosphorylate *Drosophila* KHC at the S314 residue in the α6 helix interfacing the head and the neck-linker domain (Banerjee et al., 2021). The phosphomimetic S314D and the phosphodeficient S314A substitutions severely affected the motor function. Although it did not detach the motor from the microtubule, the ATPase activity and microtubule gliding *in vitro* were significantly reduced. The mutations also affected the axonal transport of mitochondria in *Drosophila* (Banerjee et al., 2021). The neck-linker domain moves to a significant extent during the ATPase cycle to generate the force along microtubule during the ATPase cycle (Rice et al., 1999; Vale and Milligan, 2000), which is likely to transiently increase tension on the α6 segment during each stepping cycle (Qin et al., 2020). Therefore, the phosphorylation of the S314 could potentially alter the helix packing and change the overall dynamics of the neck-linker movement.

**FIGURE 1:**
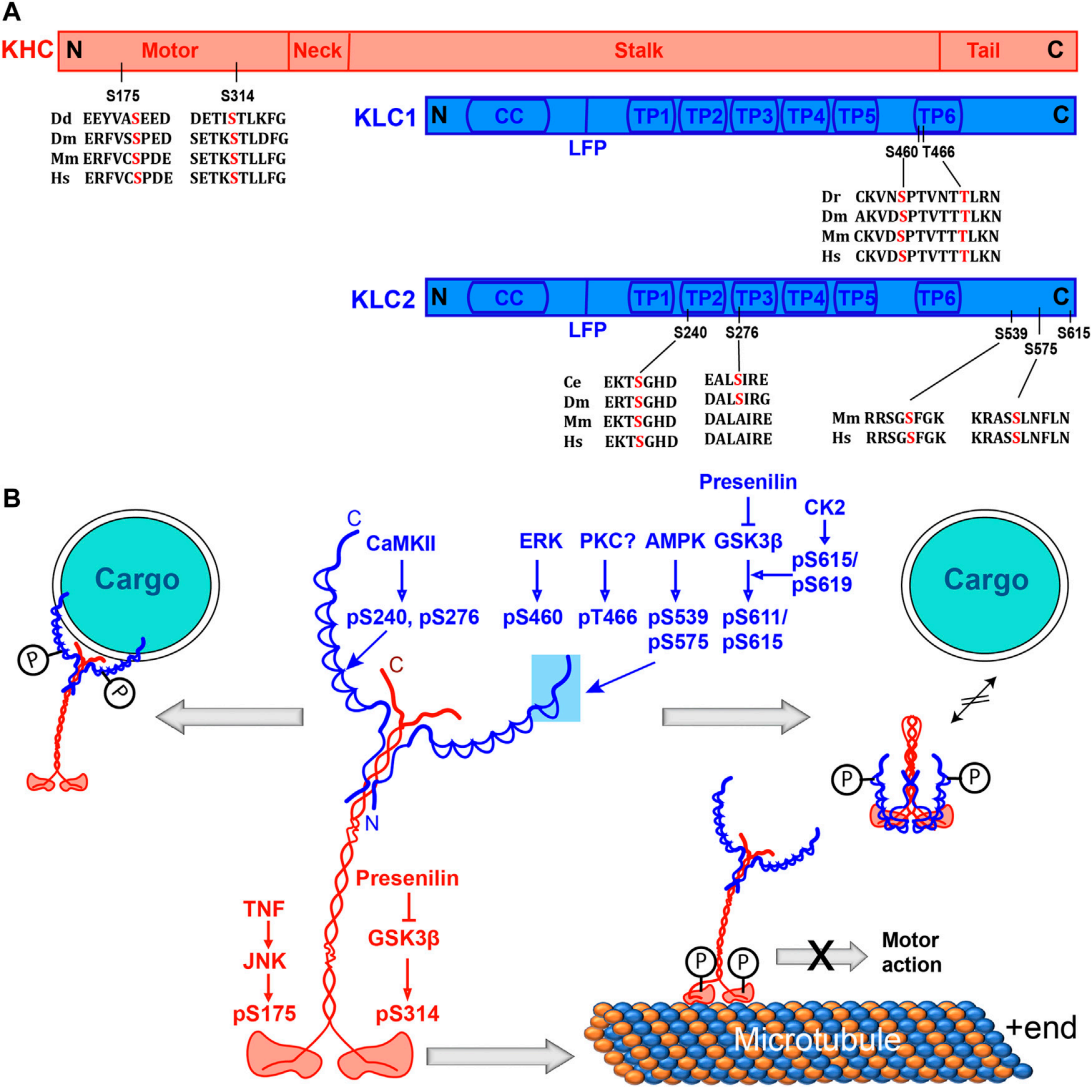
Phosphorylation sites on Kinesin-1 and their effects on the motor’s function. **(A)** Domain organizations of KHC and KLC subunits with relative positions of phosphorylation sites and alignments of the surrounding amino acid sequences from different species. Dd, Dr, Dm, Mm and Hs represent *Dictyostelium*, Zebrafish, *Drosophila*, mice, and humans. **(B)** Schematics indicate the structure of the Kinesin-1 holoenzyme and illustrate the effects of phosphorylation of different serine residues. Phosphorylation of the C-terminal part of KLC by ERK, PKC, AMPK and GSK3β, respectively, disrupts motor-cargo interactions. Phosphorylation at the N-terminal part of KLC by CaMKII recruits Kinesin-1 to GLR-1 vesicles and increases its transport. The GSK3β and JNK–dependent phosphorylation at two different resides in the motor domain disrupts the motor activity. The phosphorylation by JNK was also shown to stabilize autoinhibited conformation. Abbreviations: AMPK, Adenosine Monophosphate-activated protein kinase; CC, Coiled coil, CK2, Casein Kinase 2; cAMP, cyclic Adenosine Mono Phosphate; ERK, Extracellular Receptor Kinase; FEZ1, Fasciculation and elongation protein zeta 1; GSK3β, Glycogen Synthase Kinase 3β; JIP, cJUN interacting Protein; KHC, Kinesin Heavy Chain; KLC, Kinesin Light Chain; LFP, Leucine Phenylalanine Proline motif; PKA, Protein Kinase A; PKC, Protein Kinase C; TP, Tetra-Trico-peptide repeat; S, serine; pS, phosphoserine.

Together, these results suggest that phosphorylation of different conserved serine residues in the motor domain could attenuate the Kinesin-microtubule interaction and the ATPase rate through distinct mechanisms (Figure 1B). Further structure-function correlation studies are needed to identify the underlying mechanism and its significance *in vivo*.

## Effects of Kinesin-1 Phosphorylation on Cargo-Motor Interaction

The phosphorylation of Kinesin-1 subunits was indicated to promote and attenuate cargo association in different contexts. The outcome depends on the site of phosphorylation and the local milieu. It was also shown to stabilize the autoinhibited conformation of the motor. In one of the pioneering studies, Peter Hollenbeck and his colleagues showed that membrane-associated KHC and KLC are phosphorylated at multiple serine residues by PKA downstream of nerve growth factor (NGF) signaling in PC12 cells and chick neurons *in vivo* (Hollenbeck, 1993). The event promoted organelle transport in the neurites of PC12 cells (Lee and Hollenbeck, 1995). Although the types of kinases and target sequences were unclear, the observation indicated that KHC phosphorylation could promote cargo binding. Similarly, KLC2 phosphorylation at S575 was shown to trigger 14-3-3η binding (Ichimura et al., 2002). The 14-3-3 family of homo and heterodimeric adaptor proteins associate with a variety of other proteins, including several kinases and phosphatases (Thompson and Goldspink, 2021; Obsilova and Obsil, 2020) (Box 2). Genetic studies further showed that CaMKII-dependent phosphorylation at the N-terminal part of KLC-2 (S240 and S276) and MAPK-signaling could positively regulate an AMPA-receptor subunit (GLR-1) transport in *C. elegans* (Hoerndli et al., 2015; Hoerndli et al., 2022). Together, these findings suggested that site-specific phosphorylation by a distinct set of kinases could activate Kinesin-1 in specific contexts and promote intracellular transport (Figures 1A,B).

A KHC phosphorylation was also indicated to disrupt cargo association. Ca^2+^-dependent dephosphorylation of KHC by protein phosphatase-2Bβ (PP2Bβ) enhanced the glucose-dependent insulin release, whereas the CK2-dependent phosphorylation of KHC inhibited the vesicle transport by Kinesin-1 in these cells (Donelan et al., 2002). Thus, KHC dephosphorylation was suggested to promote insulin secretion by moving β-granules to the plasma membrane in the pancreatic β-cells after insulin stimulation. Consequently, a yeast two-hybrid screen identified KIF5C/KHC as a direct binding partner of the CK2α’ (Schäfer et al., 2009), and CK2 activation by Amyloid β (Aβ) oligomers in the axon increased KLC phosphorylation, which released Kinesin-1 from the membrane and disrupted the fast axonal transport of membrane-bound organelles in squid axoplasm (Pigino et al., 2009). These results indicate that KHC and KLC phosphorylation could also decrease cargo association and transport.

Consistent with this notion, KLC phosphorylation by several kinases was found to disrupt cargo association (Figure 1B; Table 1). One of the pioneering reports suggested that KLC phosphorylation by PKA could reduce its affinity for purified synaptic vesicles (Sato-Yoshitake et al., 1992). Similarly, KLC phosphorylation by the GKS3β, enriched at the membrane delivery sites near the growing neurite tips, released Kinesin-1 from membrane-bound organelles (Morfini et al., 2002). GKS3β can potentially phosphorylate at least two serine residues (S611 and S615) after a priming phosphorylation by Casein Kinase 2 (CK2) at the C-terminal region of KLC (Morfini et al., 2002). It was shown that Presenilin (PS), the catalytic subunit of γ-secretase, could sequester GKS3β, preventing the kinase from binding and phosphorylating Kinesin-1 in axons (Banerjee et al., 2018). Accordingly, PS-1 knockout and gene mutation increased the levels of active GKS3β in neurons, which predominantly phosphorylated KLC and caused severe neuropathy in the mouse Alzheimer’s model (Pigino et al., 2003). Also, phosphorylation at multiple sites (S539 and S575) of human KLC2 by the Adenosine Monophosphate-activated protein kinase (AMPK) in neurons disrupted the association between the p85 regulatory subunit of PI3K and KLC2, attenuating the Phospho-Inositol-3-Kinase (PI3K) transport to the neurite tips by Kinesin-1, which is essential for neurite growth (Amato et al., 2011). Hence, phosphorylation at the C-terminal of KLC appears to impart an opposite effect from that of the N-terminal region. The structural and biochemical basis of this divergence is unclear. Also, phosphorylation of each residue affected the interaction with a specific adaptor/cargo.

This selective disruption of protein binding due to residue-specific phosphorylation can help to discriminate between cargoes and adaptors and regulate the respective transport. For example, vesicles carrying Amyloid Precursor Protein (APP) can associate with two different Kinesin-1 adaptors, Alcadeinα/Calsyntenin1 (Alcα/Clstn1) and the JNK interacting protein JIP1. Kinesin-1 association with Alcα/Clstn1 at the post-Golgi vesicles is suggested to regulate the APP transport in the axons (Konecna et al., 2006; Araki et al., 2007; Ludwig et al., 2009; Vagnoni et al., 2012). Alcα/Clstn1 was shown to compete with the JIP1 for KLC binding (Araki et al., 2007) and modulate the speed of APP vesicle transport (Chiba et al., 2017; Chiba et al., 2014). Phosphorylation of the S460 residue of KLC1 (Figure 1A) by Extracellular Receptor Kinase (ERK) specifically weakened its interaction with Alcα/Clstn1 and inhibited Clstn1-dependent APP transport in cultured rat cortical neurons (Vagnoni et al., 2011). A relatively high level of the S460 phosphorylation was reported in the frontal cortex of Alzheimer’s patients (Mórotz et al., 2019). Whereas phosphorylation at the T466 of KLC1, which progressively increased in mouse brain with ageing, disrupted JIP1 binding and reduced APP transport in CAD cells (Chiba et al., 2017). Both the residues are highly conserved, and the rate of APP-vesicle transport associated with JIP1 was estimated to be higher than those associated with Clstn1 (Araki et al., 2007). Hence, this phosphorylation-dependent adaptor-switching could determine the APP distribution kinetics in axons in different contexts.

Besides, KLC phosphorylation and the absence of cargo can stabilize the autoinhibited conformation of Kinesin-1. KLC associates with the KHC stalk through the N-terminal coiled-coil domain and KLC-KHC interaction has been implicated in both activation and inactivation of the Kinesin-1 dependent transport (Gindhart et al., 1998; Verhey et al., 1998). KLC forms an autoinhibited, folded conformation involving the LFR motif and TPR domains (Yip et al., 2016). Association of the autoinhibited KLC with KHC further stabilizes the fold-back inhibitory conformation of the motor subunit (Figure 1B). A competitive interaction with the tryptophan-acidic (WD) motifs of the cargo/adaptor proteins is suggested to disengage the LFR motif, which would then associate with the positively charged tail domain of KHC, relieving the motor inhibition (Yip et al., 2016). Thus, phosphorylation of the conserved TPR6 motif and the flexible C-terminal part of KLC that disengages the cargoes/adaptors from the motor complex could revert Kinesin-1 to an inhibited state (Figure 1B). Interestingly, the C-terminal part, containing S575, S606-616, and S619 residues, is unique to the mammalian KLCs (Figure 1A), indicating that these regulations evolved late. The data also suggests that the distribution of electrostatic charges in the C-terminal end of the KLC could play a vital role in regulating motor-cargo association. This model, however, does not explain how phosphorylation of KHC and the N-terminal part of KLC could increase the cargo association and motor activation as suggested by some other experiments (Lee and Hollenbeck, 1995; Matthies et al., 1993).

To summarize, the phosphorylation of Kinesin-1 appears to both promote and inhibit the interactions with cargoes and adaptors (Figure 1B). The phosphorylation of the C-terminal domain of KLCs disrupts cargo binding and stabilizes the autoinhibited conformation of the motor, whereas phosphorylation of conserved serine residues at the N-terminal part of KLC and unknown KHC sites could facilitate adaptor binding and cargo transport. Besides, not all potential phosphorylation sites on Kinesin-1 subunits are likely to be phosphorylated *in vivo*. For example, although the consensus AMPK target sequences at T693 and S520 in KHC and KLC, respectively (Rutter and Hill, 2006), were phosphorylated by the enzyme *in vitro*, the experimental analysis failed to identify the effect of this phosphorylation on insulin vesicle movement inside a cell (McDonald et al., 2010). Many of these contrasting reports are obtained from diverse cell and tissue types. Therefore, a tissue-specific comparative experimental analysis is needed to understand the basis of this diversity.

## Phoshoregulation Through the Adaptors of Kinesin-1

Kinesin-1 binds to cargoes through several different adaptors (Box 2). Phosphorylations of Kinesin adaptor proteins are equally crucial for regulating cargo association and motor activation (Table 2). The idea is well understood for the cJUN N-terminal Kinase (JNK) interacting proteins (JIP1-4), which can independently bind to both KLC (Bowman et al., 2000; Verhey et al., 2001) and KHC (Sun et al., 2011; Fu and Holzbaur, 2013). Depending on its location, the JIP phosphorylation resulted in distinct effects. For example, JIP-1 phosphorylation at the conserved S421 by JNK, downstream of DLK (Nihalani et al., 2003), facilitates interaction with the KHC tail and activates the motor function *in vitro* (Table 2). Whereas a dephosphorylated JIP1 binds to p150^Dynactin^, switching the movement of APP vesicles in axons (Fu and Holzbaur, 2013). Thus, adaptor phosphorylation is suggested to regulate motor recruitment and transport direction. The presence of KLC harnesses JIP1 releasing the KHC-tail, which induces autoinhibition. In this context, Fasciculation and Elongation protein Zeta-1 (FEZ1) binding to the KHC-tail keeps the motor active (Blasius et al., 2007).

Although the specific mechanism is unclear, genetic analysis showed that the upstream kinases MAPKKK (DLK/Wnd) and MAPKK (MKK7/Hep) bind to APLIP1/JIP1 and inhibit JIP1-KLC interaction in *Drosophila* (Horiuchi et al., 2007). It was suggested that either JNK (Basket) or MKK7 (Hep) could phosphorylate JIP1. The JIP1 phosphorylation at T103 by JNK, and possibly by some other kinases, promoted JIP-JNK interaction, dissociating and activating DLK (Nihalani et al., 2001; Nihalani et al., 2003), which in turn further promoted association and activation of both JNK and KLC1 with JIP1. Similar phosphorylation of JIP3 by the MAPKKK/ASK1 is suggested to regulate its interaction with JNK3 (Matsuura et al., 2002). This phosphorylation-dependent feedback system is suggested to restrict JNK diffusion and possibly localise its activity to promote independent transport of apparently unrelated classes of endocytic compartments in axons (Abe et al., 2009; Sun et al., 2011; Huang et al., 2011).

**FIGURE 2:**
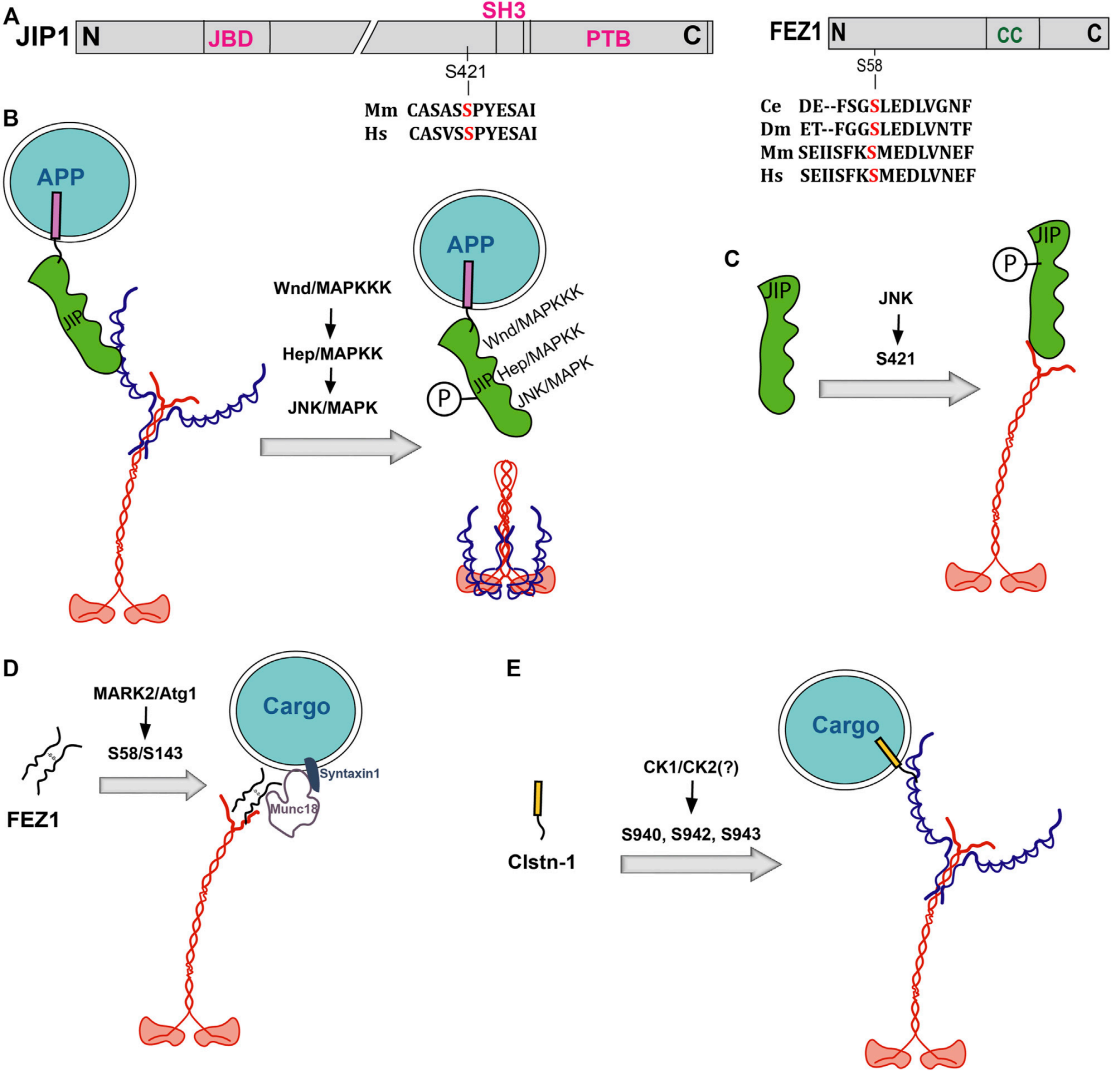
Effect of phosphorylation on Kinesin-1 adaptors. **(A)** Domain organizations of JIP1 and Fez1 with relative positions of phosphorylation sites and alignments of the surrounding amino acid sequences from different species. Ce, Dm, Mm and Hs represent *C. elegans*, *Drosophila*, mice, and humans. **(B–D).** Schematics depict how phosphorylation of JIP1 and Fez1 affect the adaptor binding to the kinesin motor. JIP1 is phosphorylation by Hep and JNK downstream of MAPKKK signaling at some unknown site(s) disrupts association with KLC **(B)**. On the other hand, JIP1 phosphorylation by JNK at S421 enhances binding to the KHC tail and promotes APP vesicle transport in DRG neurons **(C)**. Similarly, phosphorylation of both the human and *Drosophila* Fez1 at S58/S143 by MARK2 and Atg1, respectively, enhances its binding to Kif5C/KHC tail and the Munc18-Syntaxin1 complex **(D)**, and that of Alcα/Clstn1, potentially by CK1 and/or CK2, increases KLC association **(E)**.

Adaptor phosphorylation could independently regulate motor recruitment on a cargo (Figure 2). For example, phosphorylation of multiple serine residues in the C-terminal acidic region between the WD-motifs of Alcα/Clstn1 regulates the degree of its interaction with KLC (Sobu et al., 2017). In addition, phosphorylation of several serine residues distributed along the N-terminal part of Fez1 was shown to differentially regulate the interactions between Munc18-KHC complex and Syntaxin-1 (Chua et al., 2012). In this context, two distinct kinases were found to act on a particular serine residue (Figures 2B,C). The ATG1/Unc51 kinase-dependent phosphorylation of the *Drosophila* Unc76/Fez1, which forms a complex with Munc18 and Syntaxin-1 on a distinct subset of presynaptic vesicles (Chua et al., 2012), at the S143 residue increased the adaptor’s affinity for Synaptotagmin-1 (Syt-1) and initiated Syt-1 vesicle transport by Kinesin-1 (Toda et al., 2008). Whereas phosphorylation of an equivalent serine (S58) residue of mammalian Fez1 by MARK/PAR-1 turned out to be essential for the axonal transport of presynaptic components like Synaptobrevin and Syntaxin1 (Butkevich et al., 2016). Further, MARK2-dependent phosphorylation of Fez1 was shown to recruit Kinesin-1 on a viral capsid (Malikov and Naghavi, 2017). In all these cases, the phosphorylation enhanced Kinesin-1 association with the cargo.

A converse effect was observed in the case of a tubulin adaptor of Kinesin-1—the Collapsin Response Mediator Protein-2 (CRMP-2), also known as TOAD-64, Ulip2, and DRP-2 (Kawano et al., 2005). The C-terminal domain of CRMP2 binds to the TPR domains of KLC1 and transports the Sra-1/WAVE1 complex during axon formation (Kawano et al., 2005). It also regulates the microtubule organization in *C. elegans* neurons, which is essential for the KIF5/UNC-116 dependent axonal transport of mitochondria (Chen et al., 2021). CRMP2 is phosphorylated by CDK-5, GSK3β and Rho-kinase at multiple residues at the C-terminal domain and dephosphorylated by PP2A (Nakamura F. et al., 2020). The CDK-5 and GSK3β-dependent dual phosphorylation altered the CRMP2-tubulin interaction and led to the axon growth cone collapse (Yoshimura et al., 2005; Uchida et al., 2005). It is unclear whether the phosphorylation could also regulate KLC1-CRMP2 interaction in this context. Nevertheless, the result suggests that phosphorylation can differentially regulate adaptor-cargo interaction.

A distinctly different molecular mechanism appeared to regulate the mitochondria association with Kinesin-1. The mitochondrial outer membrane protein Miro binds to KHC through Milton/TRAK (Brickley et al., 2005; Glater et al., 2006; Weihofen et al., 2009; Wang and Schwarz, 2009; López-Doménech et al., 2018). The Miro-Mitochondria interaction is regulated by PTEN-induced putative kinase 1 (PINK1) and the ubiquitin ligase Parkin (Weihofen et al., 2009; Wang et al., 2011). PINK1-dependent phosphorylation of Miro induces its degradation *via* Parkin which detaches Kinesin-1 from the mitochondrial surface and promotes a minus end-directed movement on the microtubule.

Thus, phosphorylation of the adaptor-cargo interacting domains appears to promote cargo association in the case of JIP1/3, Fez1 and Clstn-1, and disrupts the interactions with CRMP2 and Miro (Figure 2). Together, these observations indicate that adaptor phosphorylation could tune the affinity with motor, adding a new layer of logistics control.

## Phosphoregulation of Kinesin-2

**FIGURE 3:**
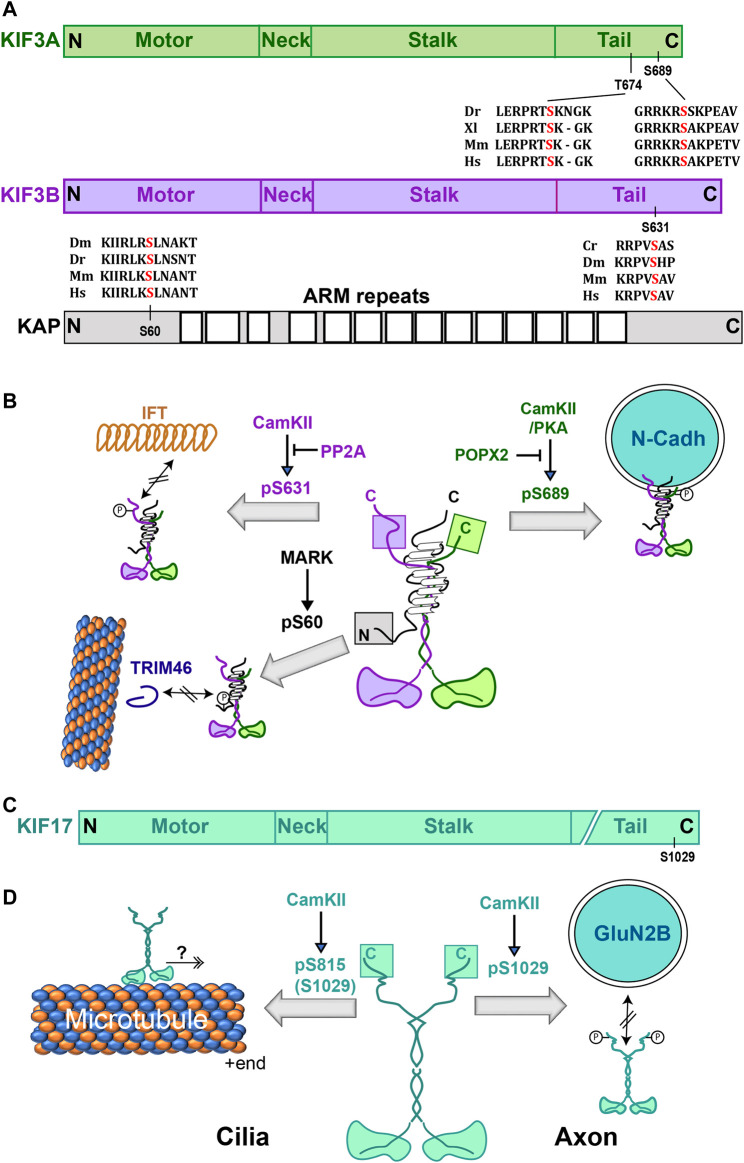
Effect of phosphorylation on Kinesin-2. **(A)** Domain organizations of mammalian heterotrimeric Kinesin-2 subunits with relative positions of phosphorylation sites and alignments of the surrounding amino acid sequences from different species. Cr, Dm, Dr, Xl, Mm and Hs represent *Chlamydomonas*, *Drosophila*, zebrafish, *Xenopus*, mice, and humans. **(B)** Schematics indicate the structure of heterotrimeric Kinesin-2 holoenzyme and illustrate the effects of phosphorylation of different serine residues. Phosphorylation of the motor subunits KIF3A and KIF3B produces opposite effects. The KIF3A (S689) phosphorylation increases association with N-cadherin vesicles, whereas KIF3B(S631) phosphorylation disrupts interaction with the IFT particles. The KAP3(S60) Phosphorylation by MARK2 reduced the Kinesin-2 affinity for TRIM46 and microtubule. **(C)** Domain organization of the mammalian homodimeric Kinesin-2 subunit with the relative position of the CaMKII phosphorylation site and alignment of the surrounding amino acids from different species. Dr, Mm and Hs represent *Drosophila*, mice and humans, respectively. **(D)** Schematics indicate the structure of the homodimeric Kinesin-2 holoenzyme and illustrate the effects of phosphorylation of different serine residues. Mouse Kif17(S1029) phosphorylation by CaMKII dissociates vesicles carrying the GluN2B receptor subunit. Phosphorylation on an equivalent residue of zebrafish KIF17(S815) promoted motor entry into the photoreceptor cilia. Abbreviations: ARM, Armadillo-like repeats; CaMKII, Ca^2+^/Calmodulin-Dependent Protein Kinase II; IFT, Intraflagellar Transport; KIF3A and KIF3B, heterotrimeric Kinesin-2 motor subunits α and β; KAP, Kinesin Accessory Protein; Kif17, homodimeric Kinesin-2 motor subunit γ; MARK2, Microtubule Associated Regulatory Kinase 2; PP2A, Protein Phosphatase 2A; POPX2, PP2C family phosphatase; TRIM46, Tripartite motif family protein.

Phosphorylation of multiple serine residues in the tail domains of Kinesin-2 motor subunits was shown to regulate the cargo-motor interactions (Figure 3; Table 1). In the case of the heterotrimeric Kinesin-2, phosphorylation of each tail imparted distinctive effects (Figures 3A,B). For example, phosphorylation of Kif3A at the conserved S689 by PKA and the partly conserved T694/S698 by CaMKIIa increased the Kif3A-dependent N-cadherin transport in the neurites of cultured mouse hippocampal neurons (Ichinose et al., 2015). The dual phosphorylation enhanced the interaction between N-cadherin and Kinesin-2, but it did not appear to affect the activity of purified Kinesin-2. An independent study further showed that dephosphorylation of pS690 of human Kif3A (equivalent to S689 of mouse Kif3A) by a PP2C family phosphatase, POPX2, could disrupt both the N-cadherin and β-catenin transport in NIH3T3 cells (Phang et al., 2014). However, contrary to data presented by Ichinose et al. (2015), Phang et al. (2014) suggested that CaMKII could phosphorylate the S690 as well. Regardless of this discrepancy, both studies suggest that the phosphorylation of the Kif3A tail domain is likely to enhance specific cargo interaction with heterotrimeric Kinesin-2 in the cytoplasm (Figure 3B). These studies also highlighted the compartment-specific effects of CaMKII-dependent phosphorylation of heterotrimeric Kinesin-2. On the other hand, the CrCDPK/CaMKII-dependent phosphorylation at a conserved S663 of the FLA8 (Kif3B orthologue) of *Chlamydomonas* at the tip of regenerating flagella released the soluble IFT-B particles from the Kinesin-2 complex and facilitated the motor turnover (Liang et al., 2014). The levels of FLA8 phosphorylation, controlled by the PP2A family of phosphatases, CrPP1 and CrPP6, determine the ciliary length (Liang et al., 2018). Thus, it suggested that the phosphorylation could negatively regulate the cargo-motor interaction in the cilia.

Phosphorylation requires an association between the enzyme and substrate. Accordingly, both MLK2 and MLK3 were found to interact with Kinesin-2 subunits using a yeast 2-Hybrid assay (Nagata et al., 1998). The C-terminal fragment of MLK2 is associated with the tail domain of Kif17, the human orthologue of *C. elegans* Osm3, as well as the Kif3A and Kif3B. The effects of these interactions are not known. In addition, the non-catalytic C-terminal domain of Ciliogenesis associated kinase 1 (CILK1), also known as Intestinal Cell Kinase (ICK), was shown to associate with the IFT-B complex along with Kif3A (Nakamura K. et al., 2020) and phosphorylate a highly conserved T672 residue in the human Kif3A tail (Oh et al., 2019), and an equivalent T674 residue of mouse Kif3A, at the ciliary tip (Chaya et al., 2014). It was proposed that the ciliary tip localization of ICK could facilitate the IFT to transition from anterograde to retrograde. Although ICK was indicated to be critical for cilia assembly in certain types of cells *in vivo* (Moon et al., 2014), Kif3A (T674A) mutation did not significantly affect the embryonic development and cilia growth in mouse embryonic fibroblast cells (Gailey et al., 2021). Therefore, it is unclear whether this particular phosphorylation could affect cargo-motor interaction or motor activity.

Further, reports indicated that phosphorylation of the heterotrimeric Kinesin-2 accessory protein, KAP, could also alter cargo-motor interaction. The tripartite motif (TRIM) family protein TRIM46 has a unique microtubule crosslinking activity essential for the organization of the axon-initial segment (AIS) (Van Beuningen et al., 2015), which directs the movement of axon-specific cargoes (Nakata and Hirokawa, 2003; Nakata et al., 2011; Morikawa et al., 2015). The phosphorylation at the conserved S(T)23 and S60 residues of KAP3 by the Microtubule-Associated Regulatory Kinases (MARK1/2) reduced the KAP3-TRIM46 association in the cell body and dendrites of mouse hippocampus neurons (Ichinose et al., 2019). Loss of Kif3B and TRIM46 altered the microtubule polarity in the AIS in tissue-cultured cells (Ichinose et al., 2019; Van Beuningen et al., 2015), and an independent study suggested that loss of Kinesin-2 function also disrupts the movement of soluble choline acetyltransferase across AIS in *Drosophila* (Sadananda et al., 2012). The result suggests that KAP phosphorylation would disrupt the cargo association (Figure 2B). Similarly, phosphorylation of the human KAP orthologue, identified as Smg GDS associated protein (SMAP) using a yeast 2-hybrid screen (Shimizu et al., 1996), by the v-Src kinase reduced its affinity for the SmgGDS in cell-free systems. However, *in vivo* effect of this phosphorylation is still not known. Interestingly, the MARK1/2 phosphorylation targets are located in the flexible N-terminal region of KAP3, which is also suggested to associate with the coiled-coil stalk of heterodimeric motor subunits (Doodhi et al., 2009). Therefore, phosphorylation could also potentially alter the interaction with the motor.

For homodimeric Kif17, CaMKII-dependent phosphorylation of S1029 at the tail domain in dendrites disrupted its interaction with the scaffold protein Mint-1, which associates with vesicles carrying the NMDA receptors subunit GluN2B (Guillaud et al., 2008; Yin et al., 2012). Whereas the phosphorylation of S815 residue of zebrafish Kif17, which is equivalent to the S1029 of mouse Kif17, increased disc shedding and the motor turnover in the cone photoreceptor outer segment (Lewis et al., 2018). The S815 phosphorylation was suggested to facilitate Kif17 entry into the cilia. These results showed how phosphorylation at equivalent positions could produce contrasting outcomes in different subcellular compartments, such as the dendrites and cilia (Figure 2D).

In summary, the tail phosphorylation of the Kinesin-2 family motors appeared to regulate type-specific cargo release and compartment-selective activation. Kinesin-2 tails are natively unfolded and directly bind to several soluble (Lolkema et al., 2007; Dishinger et al., 2010; Sadananda et al., 2012; Girotra et al., 2017; Dishinger et al., 2010) and vesicle-associated cargoes (Yin et al., 2012; Dey et al., 2017; Jana et al., 2021). Therefore, unlike Kinesin-1, phosphorylation of the tail domains of Kinesin-2 motor subunits could play a predominant role in regulating cargo interactions. Also, we noted that almost all the phosphorylation motifs are conserved within the heterotrimeric Kinesin-2 family, indicating functional conservation of the phosphorylation events (Figure 2; Table 1). Compared to Kinesin-1, our understanding of the Kinesin-2 structure and cargo-motor interaction is still rudimentary (Webb et al., 2020). Future research in this area is required to understand how phosphorylation of the same region could produce divergent effects in different subcellular compartments. Besides, the role of phosphorylation on Kinesin-2 motor activation/inhibition is not known, although several putative motifs are predicted in the motor domain.

## Phosphoregulation of Kinesin-3

**FIGURE 4:**
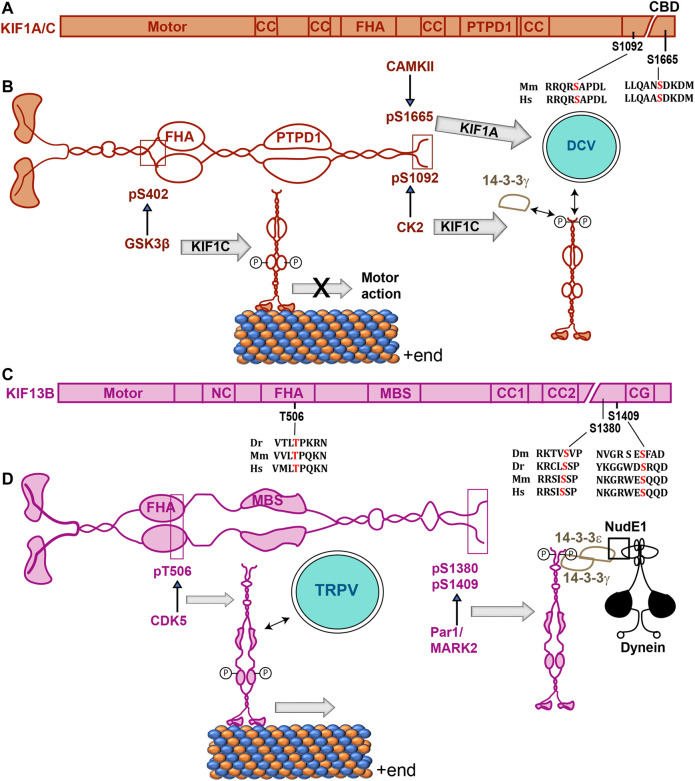
Phosphorylation sites of Kinesin-3 isoforms and their effects on their function. **(A,C)** Domain organizations of mammalian Kinesin-3 subunits with relative positions of phosphorylation sites and alignments of the surrounding amino acid sequences from different species are indicated on the primary structures. Dm, Dr, Mm and Hs represent *Drosophila*, zebrafish, mice, and humans. **(B,D)** Schematics indicate the structure of Kinesin-3 family proteins Kif1A/C **(B)** and Kif13B **(C)**, respectively, and illustrate the effects of phosphorylation of different serine residues. CaMKII-dependent phosphorylation of Kif1A increased its association with DCV, and CK2-dependent phosphorylation of Kif1C promoted association with 14-3-3γ **(B)**. In the case of Kif13B, FHA domain phosphorylation by CDK5 enhanced association with the vesicles carrying TRPV receptors, and MARK2/Par1-dependent phosphorylation in the tail domain promoted association with the dynein motor complex through 14-3-3γ/ε heterodimer **(D)**. Abbreviations: CC, coiled-coil; CDK5, Cyclin-Dependent Kinase 5; CG, CAP-Gly domain; CK2, Casein Kinase 2; DCV, Dense Core Vesicle; FHA, Fork Head Associated domain; MBS, MAGUK binding domain; NC, neck coil; PTPD1, Protein Tyrosine Phosphatase D1 binding domain; TRPV, Transient Receptor Potential Vanilloid; T-, threonine; pT, Phosphothreonine.

Kinesin-3 is a highly diverse family of motors consisting of several conserved sub-families (Box 1), and distinct target-specific effects of phosphorylation were observed (Table 1). Phosphorylation of the stalk and the tail domains of Kinesin-3 motors is suggested to block motor activity and modulate cargo binding (Figure 4). The results are summarized according to the Kif1A, Kif13 and Kif16 subfamilies in the following subsections.

BOX 1 **The long-range Kinesins.**

**Kinesin 1:** It is also known as the conventional Kinesin. The motor was the first member of the Kinesin family to be discovered (Vale et al., 1985; Yang et al., 1988). The Kinesin holoenzyme was purified as a heterotetramer of two identical heavy and light chains (Bloom et al., 1988). The Kinesin Heavy Chain (KHC) contains the conserved N-terminal motor/head domain, which is followed by a flexible neck, a coiled-coil stalk and natively unfolded tail domains (Figure 1A). The N-terminal coiled-coil domain of the Kinesin Light Chain (KLC) associates with the C-terminal part of the coiled-coil stalk of KHC. Six conserved tetra-trico-peptide repeats (TPR) motifs in the middle and a flexible C-terminal domain of KLC binds to adaptors and cargoes (Figure 1A). Auto-inhibition of the kinesin motor through the head-tail association was also studied first time using Kinesin-1 (Hackney and Stock, 2000). It was suggested that the association between the IAK motif of the tail domain and the P-loop region of the head domain could prevent the ADP-ATP exchange and arrest the motor function *in vivo* (Kaan et al., 2011). A cargo association and/or binding with KLCs could relieve this auto-inhibition. Both KHC and KLC were purified in multiple phosphorylated forms from the bovine brain (Matthies et al., 1993) and chick neurons (Hollenbeck, 1993).

**Kinesin-2:** This family of motors consists of both the heterotrimeric and homodimeric members. The heterotrimeric Kinesin-2, represented by the mammalian Kif3A/B and Kif3A/C, are highly conserved from Chlamydomonas to humans. The heterotrimeric Kinesin-2 holoenzyme, purified from several organisms, consists of two distinct motor subunits (Kif3A/2α and Ki3B/2β) and accessory protein KAP3/2κ (Figure 2A); (Scholey, 2013). Each motor subunit has a similar domain organization as KHC with a relatively shorter stalk domain. Genetic studies indicated that the accessory subunit is essential for Kinesin-2 functions (Sarpal et al., 2003; Mueller et al., 2005). KAP, suggested initially as the universal cargo adaptor, is also shown to associate with the tail domain of the vertebrate and *C. elegans* Kinesin-2 motor subunits (Wedaman et al., 1996; Yamazaki et al., 1996). However, studies with the *Drosophila* and human orthologs have shown that KAP could bind to the C-terminal part of the coiled-coil stalk domain of the heterodimeric motor (Doodhi et al., 2009) and harness the motor subunits together (Ahmed et al., 2020). The heterotrimeric Kinesin-2 is involved in cilia formation and transport into the cilium (Scholey, 2013; Webb et al., 2020). It is also implicated in the axonal transport of a range of different types of presynaptic proteins (Ray et al., 1999; Sadananda et al., 2012; Kulkarni et al., 2017) and endosomal derivatives (Tuma et al., 1998; Brown et al., 2005; Dey et al., 2017). The homodimeric Kinesin-2, represented by the mammalian Kif17 (2γ) and Osm3 of *C. elegans*, is also implicated in the receptor transport and cilia assembly (Scholey 2013).

**Kinesin-3:** Represented by Kif1, Kif13, Kif14, Kif16, and Kif28 in mammals (Miki et al., 2005; Siddiqui and Straube, 2017), these are induced homodimeric motors that are engaged in superfast, long-range transport of presynaptic vesicles and mitochondria (Hammond et al., 2009; Soppina et al., 2014). Mutations in these motors are associated with hereditary neurodegenerative disorders (Gabrych et al., 2019). Apart from a conserved N-terminal motor domain and a partly conserved phosphothreonine (pThr)-specific forkhead-associated (FHA) domain in the middle, the Kinesin-3 family motors have highly diverse domain compositions (Miki et al., 2005; Siddiqui and Straube, 2017). For example, the Kif1A and Kif16 family contains several coiled-coil domains (exceptions Kif28P and Kif16A), and the Kif13 family contains a MAGUK family guanylate kinase (GK) protein binding (MBS) domain. All of them contain a variable C-terminal region of unknown structure. Hence the regulation mechanisms of these motors are equally diverse.

BOX 2 **The Kinesin adaptors.**

**CJun N-terminus kinase (JNK)-interacting proteins (JIP1-4):** JIPs are scaffolding proteins containing a JNK-binding domain at N-terminus and protein-protein interaction domains, such as Src homology 3 (SH3) domain at their C-terminus. JIP family comprised of four genes encoding for JIP1-4. JIP1, most studied for its role as the scaffolding protein in the MAPK signalling cascade, binds to MLK, MKK7 and JNK (Whitmarsh et al., 1998). A yeast 2-hybrid screen identified JIP1, JIP2 and JIP3 as KLC interacting partners (Verhey et al., 2001). Later, it was found that JIP4, which is more similar to JIP3 than JIP1 and JIP2, also interacts with KLC (Nyaya et al., 2005). JIP1 and JIP-2 bind to Kinesin-1 through their phosphotyrosine-binding (PTB) domain at the C-terminal region (Satake et al., 2013), whereas JIP-3 and JIP-4 bind to KLC through a leucine-zipper domain (Nyaya et al., 2005; Cockburn et al., 2018). JIP-1 acts as an adaptor for APP and APOER2 transport by Kinesin-1, while JIP-3 helps recruit Kinesin-1 onto TrkB receptor carrying vesicles inside neurons (Huang et al., 2011).

**Fasciculation and elongation protein zeta 1 (Fez1):** Fez1 is the mammalian orthologue of *unc-76* (McIntire et al., 1992)*.* FEZ1 contains a coiled-coil domain towards its C-terminus through which it can interact with different proteins. The N-terminus region has three glutamic-acid rich regions that help in FEZ1 dimerization, which can also form a heterodimer with its homologue FEZ2 (Assmann et al., 2006). FEZ1 interacts with the inhibitory tail domain of KHC and relieves the motor from its fold-back conformation. (Blasius et al., 2007). It also helps in the recruitment of Kinesin-1 to certain cargoes, for example, KIF5C to the Syntaxin 1a (Stx1a) and Munc-18 complex (Chua et al., 2012) and KIF5A/B to HIV capsid envelope (Malikov et al., 2015).

**Calsyntenin 1 (Clstn1):** Clstn1 is a transmembrane protein belonging to the cadherin superfamily. Vertebrates have three Clstn genes (Clstn1-3). Clstn-1 binds to the TPR domain of KLC through W-acidic (WD) peptide motifs present in its cytoplasmic domain (Konecna et al., 2006) and recruits Kinesin-1 on vesicles carrying amyloid precursor protein (APP) (Vagnoni et al., 2012) and other proteins (Araki et al., 2007). Recent studies suggest that Clstn1 also has a role in organizing microtubule dynamics during axon maturation (Ponomareva et al., 2014; Lee et al., 2017).

**SifA-Kinesin interacting protein (SKIP):** SKIP binds to SifA, an effector protein secreted by the bacterium *Salmonella typhimurium* that helps in the multiplication and virulence of the bacterium in the host cell (Dumont et al., 2010). SKIP also recruits Kinesin-1 to lysosomes and mediates their anterograde dispersion inside the cell (Sanger et al., 2017). SKIP contains a RUN domain followed by W-acidic (WD) motifs at the N-terminus and a pleckstrin homology domain at the C-terminus. The C-terminus pleckstrin homology domain of SKIP is required to bind SifA. Interestingly, the same WD motifs at the N-terminus of SKIP are required to bind the KHC tail and KLC.

**Collapsin response mediator protein (CRMP):** CRMP2 associates with microtubule. CRMP2 interacts with heterotetrameric Kinesin-1 through the KLC subunit and acts as an adaptor for the Kinesin1-tubulin complex (Kawano et al., 2005). There are five homologous CRMP genes, of which CRMP2 (previously known as CRMP62) shares significant homology with the *unc33* gene of *C. elegans*. CRMP2/Unc-33 helps in Kif5A/Unc-116 mediated transport of mitochondria in *C. elegans* neurons by organizing stable microtubule bundles around the cell body and along the axons (Chen et al., 2021). CRMP2 also aids in the polarized sorting of proteins in the neurons by regulating Kif1A (Maniar et al., 2012).

**Milton/TRAK:** Milton recruits KHC on mitochondria in association with an atypical GTPase called Miro and drives the mitochondria transport in axons and dendrites (Stowers et al., 2002; Guo et al., 2005). KHC binds to the N-terminus coiled-coil domain, and Miro binds to the adjacent C-terminal part of Milton (Glater et al., 2006; Smith et al., 2006). Mammals code for two Milton/TRAK isoforms—TRAK1 and TRAK2. TRAK1 interacts with Kinesin1/KIF5A and dynein-dynactin complex and steers the mitochondrial transport into the axon, while TRAK2 steers the mitochondrial transport towards the dendrites by interacting with dynein (van Spronsen et al., 2013). TRAK2/GRIF1 is also associated with the γ-Aminobutyric acid, type A (GABA) receptor (Beck et al., 2002).

**Protein tyrosine phosphatase non-receptor type 21 (PTPN21):** PTPN21, also known as PTPD1, is a peripheral membrane protein that links the membrane-associated focal adhesion proteins to cytoskeletal proteins (Carlucci et al., 2008). It has an N-terminus four-point one-ezrin-radixin-moesin (FERM) domain and a catalytic phosphatase domain at the C-terminus. PTPN21 can interact with Kinesin-3 subfamily proteins Kif1A and Kif16B through the FERM domain (Dorner et al., 1998; Carlucci et al., 2010). PTPN21 binding can activate Kif1A by relieving the autoinhibited conformation (Siddiqui et al., 2019).

**Hook3:** Humans have three Hook proteins- Hook1, 2, and 3. Hook proteins have three distinct domains, an N-terminus microtubule-binding domain, a central coiled-coil domain and a C-terminus unstructured domain. Hook1 and -3 can interact with the dynein-dynactin complex and increase the processivity of dynein-based transport (Olenick et al., 2016). Hook3 can bind to the stalk domain of the Kinesin-3 motor KIF1A and relieve it from its autoinhibited state (Siddiqui et al., 2019). Thus, hook protein acts as an adaptor for both Dynein and Kinesin; it also helps in recruiting them to early endosomes (Bielska et al., 2014).

**Centaurin:** Centaurins are characterized by one or more pleckstrin homology (PH) domains, through which they can bind to several phospholipids and an Arf-GTPase domain. There are five different isoforms in Centaurin family of proteins- α1, α2, β, σ and ϒ. Centaurin α1 (CENTA1) has two PH domains. CENTA1 preferentially binds to phosphatidylinositol-3,4,5 triphosphate (PIP3) and phosphoinositol-3,4-biphosphate (PI(3,4)P_2_) and the FHA domain of KIF13B through its PH domains (Tong et al., 2010). CENTA1 is also important for recruiting KIF13A to AMPAR vesicles (Gutiérrez et al., 2021).

**Discs large 1 (Dlg1):** Dlg1 is a member of the membrane-associated guanylate kinase domain (MAGUK) protein family. It interacts with the MAGUK-binding stalk (MBS) domain of the Kinesin-3 subfamily protein KIF13B/GAKIN. Human disc large (hDlg) is present in two different isoforms-hDlg-I2 and hDlg-I3. hDlg has intramolecular interactions and is present in an inhibited conformation. SH2-I3-GUK segment of hDlg-I3 is required to inhibit intramolecular interactions of GAKIN and activate its ATPase activity (Yamada et al., 2007).

**Mint-1:** Mint family of proteins has three genes (Mint-1,2,3). They have a variable N-terminal domain and a conserved C-terminal domain having one phosphotyrosine-binding (PTB) sub-domains and two PDZ subdomains. The Mint-1 protein interacts with the tail domain of homodimeric Kinesin-2 KIF17 through one of its PDZ domains and acts as an adaptor for the transport of NMDA receptor subunit 2B (NR2B) into the dendrites of glutamatergic neurons (Setou et al., 2000).

**14-3-3:** The 14-3-3 family proteins generally bind to a target motif containing phosphorylated serine or threonine. Mammals express seven isoforms of 14-3-3 (β, γ, η, ε, ζ, σ, τ), while the *Drosophila* genome only codes for 14-3-3 ε and ζ. The 14-3-3 proteins are present as homo or heterodimers. The N-termini of 14-3-3 monomers form a conserved central groove that binds to phosphorylated motifs on the target proteins. 14-3-3 proteins are expressed in all types of cells. In *Drosophila* oocytes, 14-3-3 ζ interacts with a Kinesin-3 subfamily member Khc73/Kif13B, and 14-3-3 ε interacts with a dynein adaptor NudE. The 14-3-3 heterodimerization brings about a coordination between the various kinesin and dynein motor proteins required for the organization of mitotic spindles (Lu and Prehoda, 2013). Similarly, 14-3-3 η, along with Kif3A and Par-3, localizes at the tip of the cilia, which is essential for ciliogenesis (Fan et al., 2004).

BOX 3 **Major kinases that also phosphorylate Kinesin.**

**Adenosine Monophosphate-activated protein kinase (AMPK):** An S/T kinase that consists of three subunits. α subunit contains the catalytic domain, β subunit binds glycogen, and the γ subunit contains four ATP/ADP/AMP binding Bateman domains. It phosphorylates S/T residues. The phosphorylation of the T172 of α subunit by upstream kinases, such as the CaMKKβ, and increasing concentration of AMP activates the enzyme allosterically. AMPK acts as the metabolic sensor and targets a wide variety of pathways (Hardie et al., 2012; Rodríguez et al., 2021).

**Casein Kinase 2 (CK2):** A heterotetramer of two catalytic and two regulatory subunits that acts on a large variety of substrates containing the consensus “S/T-X-X-E/D/pS/pY” motif except for Casein. The enzyme activity is maintained at a low level in normal cells except during embryogenesis and in cancerous tissue, where the levels are much higher. It controls protein synthesis by upregulating the rRNA levels through direct phosphorylation of RNA and spliceosomal machinery and targets a large variety of cytoskeletal and motor proteins, such as myosin-1, X, XVIIIa, Dynactin, Cytoplasmic Dynein Light Intermediate Chain 1 (LIC1), capping protein, Gelsolin, Septin, etc. It reduces the death/survival ratio, favours angiogenesis, and inactivates tumour suppressors in cancerous tissue. CK2 also acts as a scaffold to stabilize microtubule dynamics in cilia/flagella, spindle body, and axon-initial segment (Venerando et al., 2014; D’Amore et al., 2019).

**Ca** ^**2+**^ **/Calmodulin-Dependent Protein Kinase II (CaMKII):** It is a ubiquitous enzyme of the CaMK superfamily with four (α, β, γ, and δ) highly homologous and tissue-specific isoforms, each containing regulatory and substrate-binding catalytic sites, and involved in cellular Ca^2+^ mediates signalling. The holoenzyme consists of 12 subunits of homo and heteromeric isoform composition. The Ca^2+^-dependent Calmodulin (CaM) binding to each subunit of the holoenzyme activates the enzyme independently, and an intra and intersubunit autophosphorylation of a conserved threonine residue at the regulatory domain modulates the enzyme activity. CaMKII targets an extensive repertoire of proteins carrying the consensus S/T residues. It is extensively studied in the context of learning and memory formation, epilepsy, schizophrenia and other neuropsychiatric disorders (Bayer and Schulman, 2019; Robison, 2014).

**Cyclin-Dependent Kinase 5 (CDK5):** Homologous to the family of proline-directed serine/threonine protein kinases of the Cdc2/CDK1 family involved in cell cycle progression, the enzyme is predominantly expressed in terminally differentiated neurons. Unlike the other CDK homologues, CDK5 binds to a non-Cyclin regulatory subunit called p35/39. Cdk5 and its orthologues—Cdk5r1/2 are also expressed in non-neuronal tissues during developmental stages. Myristoylatation of the p35/39 helps to associate Cdk5 to vesicles and plasma membrane. Calpain-dependent proteolysis of p35 at the plasma membrane hyperactivates the enzyme (Pao and Tsai, 2021). The enzyme targets a large variety of proteins involved in multiple different pathways (Gao et al., 2021).

**Cyclic-AMP-dependent Proteins Kinase (PKA):** A tetrameric holoenzyme complex comprising a regulatory subunit homodimer and two catalytic subunits (R_2_:C_2_) that act as a cAMP-dependent allosteric molecular switch and transmits the second messenger signal downstream of several G-protein coupled receptors (Kim et al., 2007; Zhang et al., 2012; Taylor et al., 2012). All PKA substrates must contain a minimal RR-x-S/T motif (Moore et al., 2003). It has four regulatory and three catalytic isoforms that can freely combine in different permutations. Each combination has a unique cAMP-dependent kinetics and target specificity (Zhang et al., 2012). The A-kinase anchoring proteins (AKAPs) recruit the enzyme to a larger macromolecular complex (Newlon et al., 2001; Taylor et al., 2012). cAMP binding to the R subunits releases the C units. Phosphorylation of the R subunits could also activate PKA without cAMP (Haushalter et al., 2018).

**Glycogen Synthase Kinase 3β (GSK3β):** A CMGC (CDK, MAPK, GSK-3, CLK) family S/T kinase, originally identified in glucose metabolism downstream of insulin signaling, was later shown to target a wide variety of proteins underlying several signaling pathways such as the Wnt and Notch signaling. It binds to targets pre-phosphorylated by the priming kinases such as CKII. The phosphorylation of S9 on GSK-3β by AKT/PKB and other kinases inactivates the enzyme, whereas the Y216 phosphorylation through FYN2 and PYK2 activates it (Patel and Woodgett, 2017).

**cJUN N-terminal Kinase (JNK):** One of the three-tiered mitogen-activated protein kinase (MAPK) cascade enzymes that acts at the penultimate step in relaying the signal. It belongs to the CMGC family, which is activated by a dual threonine and tyrosine phosphorylation of the TPY motif by the MAP2K family enzymes MKK4 and MKK7 downstream of the MAK3K enzymes DLK1 (Karin and Gallagher, 2005; Zeke et al., 2016). Proteins with a consensus sequence that binds to the D-site of JNK are phosphorylated by the enzyme (Whisenant et al., 2010).

**MAP/microtubule affinity-regulating kinases (MARK1-4):** These enzyme isoforms were identified in the context of tau hyper-phosphorylation-dependent development of Alzheimer’s pathology (Drewes et al., 1997). They belong to the AMPK subfamily of the CaMK group. The enzyme, enriched in the brain, kidney and spleen, phosphorylates serine residues. Phosphorylation of a conserved threonine residue at the catalytic domain activates the enzyme, and that of a conserved serine residue inactivates the enzyme (Timm et al., 2008). MARK1/2, also known as Par1, is involved in determining cell polarity and neuronal differentiation (Kemphues, 2000). Besides Tau, the MARK targets include microtubule-associated proteins—MAP2, MAP4, doublecortin, and phosphatases regulating 14-3-3 binding.

**Protein Kinase C (PKC):** An AGC-family kinase that is generally classified as lipid-dependent serine/threonine kinase. PKC isoforms belong to three subfamilies - conventional (cPKCs; α, βI, βII, and γ), non-conventional (nPKCs; δ, ε, η, and θ), and atypical (aPKCs; ζ, ι, and λ). The N-terminal regulatory domain contains a pseudosubstrate motif that binds to the C-terminal kinase domain and inhibits the enzyme activity. The N-terminal regulatory subdomains of cPKCs bind to Di-acyl-glycerol (DAG) or phorbol 12-myristate 13-acetate ester (PMA) and anionic phospholipids in the presence of Ca^2+^. The lipid-binding recruits the enzyme onto the membrane and activates the enzyme through a structural reorganization that expels the autoinhibitory, pseudosubstrate motif from the substrate-binding pocket. The cPKC and nPKC activities are controlled by phosphatidic acid and ceramide binding to the regulatory domain and phosphorylation of a threonine residue in the activation look of the kinase domain (Steinberg, 2008). The nPKC and aPKC isoforms are also activated by tyrosine phosphorylation in the catalytic domain by heterologous enzymes, such as the Src-family kinases and adaptor binding. Distinct PKC isoforms are recruited to the plasma membrane and on the cell’s vesicular membrane and act on a specific set of substrates (Geribaldi-Doldán et al., 2019; Kawano et al., 2021).

**Src-Family Kinase (SFK):** These are tyrosine phosphorylating enzymes, homologous to the oncogene cSrc/pp60^cSrc^, and include nine members (c-Src, Yes, Fyn, Fgr, Lyn, Hck, Lck, Blk, and Yrk) (Portugal et al., 2021; Thomas and Brugge, 1997). SFKs are associated with the receptor-linked tyrosine kinases (RTKs) (Ingley, 2008), and they are highly expressed in the developing tissue as well as cancerous cells (Thomas and Brugge, 1997). Phosphorylation of a conserved tyrosine residue in the C-terminal domain by C-terminal Src kinase (CSK) or CSK-homologous kinase (Chk) maintains the enzyme in an inactivated state (Okada and Nakagawa, 1989), whereas auto/transphosphorylation of a different tyrosine residue in the kinase domain activates the enzyme (Ozkirimli and Post, 2006).

In mammalian cells, Kif1A binds to Calmodulin (CaM) in the presence of Ca^2++^, which promotes the dense-core vesicles (DCV) association (Stucchi et al., 2018). Phosphorylation at S1665 near the C-terminal end of mouse Kif1A by the CaMKII regulates the Kif1A-DCV interaction through the small GTPase Arl8 (Hummel and Hoogenraad, 2021). Mutation of an equivalent S1758 residue of the human Kif1A is associated with hereditary sensory and autonomic neuropathies (Rivire et al., 2011). Here, the MmKif1A (S1665) phosphorylation is suggested to promote the cargo association. Similarly, CK2-dependent phosphorylation at the S1092 of HsKif1C enhanced the 14-3-3*γ* interaction (Dorner et al., 1999). Since 14-3-3 proteins act as a scaffold for a large variety of proteins inside a cell, Kif1C and 14-3-3*γ* interaction could also attach the motor to other proteins. A similar interaction between 14-3-3ζ and *Drosophila* Kif13B orthologue Khc73 is suggested to harness the dynein adaptor NudE through 14-3-3ε/ζ heterodimerization downstream of the cell polarity factors Pins and Dlg1 during the spindle positioning event (Lu and Prehoda, 2013).

Complex intramolecular interactions involving the motor domain, neck-coil, the coiled-coil stalk and the FHA domain inactivate the Kinesin-3-family motor (Al-Bassam et al., 2003; Lee et al., 2004; Ren et al., 2018; Siddiqui et al., 2019). A recent study further showed that the Aβ-induced activation of GSK3β could attenuate Kif1A transport in neurites of the mouse hippocampal neurons (Gan et al., 2020). Although GSK3β phosphorylated S402 of rat Kif1A *in vitro*, it did not appear to alter the motility of an autoinhibition-deficient KIF1A, suggesting that phosphorylation of additional components may be involved in this process. In the case of mammalian Kif1C, the motor-stalk interaction is disrupted by the binding of PTPN21, a FERM-domain containing protein tyrosine phosphatase, or Hook, a cargo adaptor (Siddiqui et al., 2019). It is unclear whether the PTPN21 binding could dephosphorylate the motor or whether a phosphorylation event could regulate its association. Also, an independent study suggested that the Hook3 is redundant for Kif1C activation, but it is carried to the cell periphery, where it could act as a scaffold to recruit and activate the dynein motor (Kendrick et al., 2019). Therefore, the PTPN21 and Hook3 binding are likely to have a distinct impact on Kif1C function within the cell. Together, these data indicated that potential phosphorylation of Kinesin-3 outside the motor domain could activate or deactivate the motor.

The Kif13A/B family of homodimeric proteins are implicated in a variety of intracellular transports both in neurons and somatic cells (Siddiqui and Straube, 2017). The *C. elegans* KLP-4 and its mammalian orthologue, Kif13A, transport the AMPA receptors GLR-1 to the ganglionic synapse of the worm and GluA1 to the dendritic spines of CA1 neurons, respectively (Monteiro et al., 2012; Gutiérrez et al., 2021). The AMPAR is associated with the rat Kif13A stalk domain through centaurin-*α*1 and the Rab11 adaptor FIP1 in response to neuronal activation (Gutiérrez et al., 2021). Although genetic studies implicated the activity of Cyclin-Dependent Kinase, CDK5, in GLR-1 trafficking, the phosphorylation/dephosphorylation did not appear to regulate the GluA1-Kif13A interaction (Gutiérrez et al., 2021). Therefore, this evidence suggests that some kinases can act as cargo adaptors, or they may act on other proteins that could modulate distinct cargo-motor interactions and motor activation.

The Kif13B/GAKIN family proteins also contain an MBS domain that binds to the PDZ domain-containing MAGUK family protein Discs Large 1 (Dlg1) and the microtubule-binding CAP-Gly (CG) domain at the C-terminal end (Hanada et al., 2000; Asaba et al., 2003). Uncharacteristically, the Dlg1 guanylate kinase (GK)-domain associated with an unphosphorylated segment of the MBS domain (Zhu et al., 2016), activating Kif13B motor function (Yamada et al., 2007). MAGUK-GKs usually bind to a phosphorylated target sequence. However, in this context, the Kif13B MBS was shown to compete with phospho-LGN peptide for the Dlg1-GK binding (Zhu et al., 2016). Independent studies suggested that centaurin-α1, also known as the PIP3 binding protein (PIP3BP), could specifically bind to the unphosphorylated target sequence in the FHA domains of Kif13A and 13B to release the autoinhibition and activate PI3P vesicle transport to the neurite tips (Tong et al., 2010; Horiguchi et al., 2006). In comparison, phosphorylation of T506 within the FHA domain of mammalian KIF13B by CDK5 was shown to regulate TRPV receptor binding in CHO cells (Xing et al., 2012). Thus, phosphorylation of the FHA domain could potentially regulate selective cargo interaction and motor activation for the Kif13 family of motors. On the other hand, the unphosphorylated MBS domain could regulate the oligomerization of MAGUK-GKs and transport them to specific destinations in the cell through a competitive interaction.

Therefore, the Kinesin-3 phosphorylation, predominantly in the dimerization and tail domains, appeared to regulate the cargo-motor interaction and motor activation independently (Figure 4). These domains are also the region where several independent adaptors bind to the Kinesin-3 to facilitate the motor dimerization, which is essential for activating the processive microtubule-dependent movement and cargo association.

## Phosphorylation-Dependent Change of Transport Direction

Organelles and endosomal vesicles carrying various proteins move bidirectionally on the microtubule and actin cytoskeleton. In all these cases, Kinesins power the plus-end-directed movement on the microtubule, and almost all minus-end directed movements are powered by cytoplasmic Dynein. In many instances, mutually opposing motors are recruited on the vesicle membrane by activating various small GTPases and their adaptors (Kjos et al., 2018). For example, Rab4 activation recruits both Kinesin-2 (Imamura et al., 2003) and dynein (Bielli et al., 2001) on the early and late endosomal vesicles. There is also evidence suggesting direct interactions between Kinesin and Dynein subunits. For instance, both KLC1 and KLC2 were shown to directly bind to the Dynein Intermediate Chain (DIC) through the N-terminal part, excluding the TPR motifs (Ligon et al., 2004). Kinesin-1 and Dynein were also recruited together on mitochondria through the TRAK/Milton family adaptors (Fenton et al., 2021). Similarly, Kinesin-2 and Dynein were purified together with the melanosomes from *Xenopus* melanocytes (Deacon et al., 2003), and the Kinesin-3 isotypes were demonstrated to associate with cytoplasmic Dynein using the 14-3-3 family of adaptors (Lu and Prehoda, 2013). Finally, almost a third of the endosomal vesicles were stained with both Kinesin-1 and Dynein (Ligon et al., 2004). The biophysical analysis further suggested that the mechanical coupling between the opposing motors is essential for maintaining the processive movement of cellular organelles (Kural et al., 2005; De Rossi et al., 2017; Akhmanova and Hammer, 2010).

These observations raise an interesting question: How are the activities of opposing motors controlled to generate a net unidirectional flux? *In vitro* experiments demonstrated that the direction of a vesicle movement is stochastically determined through tug-of-war, where the numerical strength of Dynein versus Kinesin motors wins the direction (Soppina et al., 2009; Rezaul et al., 2016). However, experimental data obtained *in vivo* indicated that a type-specific activation/inactivation of the attached motors could regulate the long and processive bidirectional movements (Gross et al., 2000; Gross et al., 2002; Kunwar et al., 2011). In this latter situation, the presence of microtubule-associated proteins (Dixit et al., 2008) and phosphorylation (Serpinskaya et al., 2014) are suggested to switch the direction of the vesicle’s movement. Such regulation is often reversible and does not involve dissociating the motors from vesicles or organelles, allowing the organelle to adjust its position continuously with respect to the changing cytoskeleton architecture and local conditions. Here, we discuss a few such cases where phosphorylation of a Kinesin or activity of a specific set of kinases are identified to regulate the organelle movement.

Studies in tissue-cultured cells indicated that stimulation with the cytokine Tumour Necrosis Factor (TNF) leads to aggregation of mitochondria near the microtubule-organizing centre in the perinuclear region (De Vos et al., 1998). The treatment selectively hyper-phosphorylated KLC and reduced the Kinesin-dependent mitochondrial motility towards the cell periphery (De Vos et al., 2000) but did not appear to dissociate Kinesin from the mitochondria (De Vos et al., 1998). TNF activates several kinases downstream. In L929 cells, KLC was phosphorylated by two different kinases. Amongst these, the p38 Mitogen-Activated Protein Kinase (MAPK), activated by the mixed lineage kinase MLK2 downstream of the TNF receptor, was shown to inhibit Kinesin-1 motility and increase the perinuclear clustering of mitochondria (De Vos et al., 2000). The phosphorylation target sequences are not known. The evidence may suggest that selecting a specific signaling downstream is likely to regulate the direction of the vesicle movement inside a cell.

Similarly, a combination of Kinesin-2, Dynein and Myosin V moves melanosomes inside melanocytes (Tuma et al., 1998; Rogers et al., 1997). The Myosin V activity is required for partial dispersal and uniform distribution of the melanophores (Rogers et al., 1997), and a complete dispersal requires the heterotrimeric Kinesin-2 (Tuma et al., 1998). The movement is regulated by the kinases, PKA and PKC, and the PP2A phosphatase (Reilein et al., 1998). The PKA activation downstream of α-Melatonin Stimulating Hormone (MSH) fully disperses the melanosome granules, and melatonin/PMA activated PKC partially disperses the granules. The PP2A phosphatase activity induced melanosome aggregation. The MSH and melatonin/PMA altered Dynein subunit phosphorylation on melanosomes, but their effects on Kinesin-2 are still unclear. The PKA-dependent phosphorylation inactivated Casein Kinase 1ε (CK1ε), which in turn inactivated a kinase associated with the melanosomes and prevented the activation of Dynein by CK1ε-dependent phosphorylation of DIC (Ikeda et al., 2011). The Dynein activation by CK1ε, thus, promoted aggregation of the pigment granules in a fashion similar to that of melatonin stimulation.

Together, the evidence suggests highly divergent effects of motor subunit phosphorylation. The phosphorylation often has opposing effects on the activation of two different types of motors, such as a Kinesin and Dynein associated with the same cargo, altering the directional flux. As demonstrated with the melanosome and lysosome movements, such changes are coupled to external stimuli affecting cellular physiology.

## Discussion

The analysis revealed that a host of cytoplasmic kinases involved in signaling and metabolic regulation (Box 3) target the long-range kinesins producing highly diversified effects. Most phosphorylation targets in Kinesin-1 are conserved serine and threonine residues, with some located in unique sequences only found in mammals (Figure 1A). These observations may suggest that regulation of Kinesin-1 function is a constantly evolving mechanism. Interestingly, all the phosphorylation sites located in the tail domains of Kinesin-2 are highly conserved (Figure 3A), though most of the tail sequences are poorly conserved, suggesting that phosphoregulation is a fundamental aspect of basic Kinesin-2 biology that could have evolved earlier than the other two Kinesin families. Overall we also find that the sites of Kinesin phosphorylation and the type of kinases acting on them are highly divergent and family-specific, with a few notable exceptions such as CaMKII and GSK3β (Table 1). However, the phosphorylation targets of these two kinases are not congruent across the families (Table 1). For example, the GSK3 site is located in the KHC head domain, distinct from the Kif1A tail (Figures 1, 3). Also, the same kinase, such as GSK3β and PKA, was shown to phosphorylate both the motor (KHC) and accessory (KLC) subunits (Table 1). Each of these phosphorylation events imparted a unique effect. This diversity is essential for managing the complex logistics of intracellular transport.

The phosphorylation mainly acts as a switch. For example, phosphorylation of the KHC motor domain and the coiled-coil stalk domain of Kinesin-3 disrupted the microtubule-dependent motility and converted the motor to an inactive state. A similar effect was observed in the case of Kinesin-5, -4 and -13 family members (Garcia et al., 2009; Bickel et al., 2017; Avunie-Masala et al., 2011; Chee and Haase, 2010; Shapira and Gheber, 2016; Poser et al., 2020; Lan et al., 2004; McHugh et al., 2019). Further, the phosphorylation of the C-terminal tail domain of Kinesin-2 and -3 family members resulted in either association or dissociation of the cargo adaptors and motor activation, and Kinesin-7 tail-domain phosphorylation activated the motor (Espeut et al., 2008; Kim et al., 2010).

Amongst this diversity, one could also find some consensus features of phosphoregulation. For example, phosphorylation of KLCs at the C-terminal and the N-terminal part of KAP disrupted cargo association and transport (Tables 1, 2). Also, KLC phosphorylation at the N-terminal part and KHC phosphorylation facilitated specific cargo transport in different contexts. In comparison, phosphorylation of the Kinesin adaptors, Fez1, Clstn1, and JIP1, generally facilitated their association with Kinesin-1 and activated the motor, except for one occasion, when the JIP1 phosphorylation disrupted its association with KLC (Figure 2; Table 2). Thus, to a large extent, phosphorylation of accessory proteins and adaptors is likely to enhance cargo transport. The process is also coupled with the association of the kinase cascade. For instance, the kinases harnessed by the adaptor also modulate engagement with the JIP scaffold. Moreover, the kinases involved in Kinesins regulation have very different rate kinetics and distinct tissue-specific subcellular localizations (Box 3). In this context, the phosphorylation propensity determined by the rate kinetics of the kinases involved is likely to tune the cargo-specific flux. For example, a competition between the JNK and ERK could control the axonal APP traffic by switching between the JIP1 and Clstn1 adaptors. Therefore, how the kinase property contributes to traffic modulation would be an exciting topic of future research.

The other exciting idea that emerged from these studies is that the subcellular localization of the kinases may have a substantial impact on the logistics as it spatially segregates the on/off actions to facilitate the intracellular movements from one point to another. For instance, the localization of PINK1 with Parkin and TRAK on mitochondria is essential for regulating its distribution in the cell. Similarly, Kinesin-2 phosphorylation by CaMKII produced opposite outcomes in axons and cilia (Figure 2C). In addition, the enrichment of GSK3 at the distal ends of growing neurites dislodged membrane-bound organelles and TRPV receptors at the growing tips of neurites, facilitating neurite growth.

The phosphorylation by the same kinase often produces opposite effects on the Kinesin and Dynein. For example, ERK1/2-dependent phosphorylation of Dynein-intermediate chain, downstream of TRK signaling, recruits and activates the motor, whereas ERK phosphorylation of KLC dissociates it from Clstn1. In contrast, the GSK3β-dependent phosphorylation disrupted both Kinesin and Dynein association with a cargo. An equally diverse set of kinases are shown to act upon the dynein subunits and regulate the cargo association (Tempes et al., 2020). Evidence suggests that a complex kinase-specific regulation determines cargo transport dynamics (Gibbs et al., 2015). Some kinases and phosphatases acting on Kinesins and Dynein, such as CK2, JNK and PP2A, are also stably associated with the motor. Therefore, the motor activity resulting from their action could also establish a dynamic spatiotemporal distribution of these enzymes, which in turn could influence the overall logistics—a fascinating possibility that needs to be investigated in the future.
